# Piwil2 Suppresses P53 by Inducing Phosphorylation of Signal Transducer and Activator of Transcription 3 in Tumor Cells

**DOI:** 10.1371/journal.pone.0030999

**Published:** 2012-01-27

**Authors:** Yilu Lu, Kun Zhang, Chao Li, Youlin Yao, Dachang Tao, Yunqiang Liu, Sizong Zhang, Yongxin Ma

**Affiliations:** State Key Laboratory of Biotherapy, Division of Morbid Genomics, Department of Medical Genetics, West China Hospital, Sichuan University, Chengdu, China; Emory University, United States of America

## Abstract

Piwi proteins have been implicated in germ cell proliferation, differentiation, germline stem cell maintenance and transposon control in germline from Drosophila to mammals. The Piwi-like2 (piwil2) gene is mainly expressed in testis or embryonic cells among normal tissues but widely expressed in tumors. However, it remains to be fully determined through which mechanism piwil2 is involved in tumorigenesis. Here we report that Human piwil2, or Hili represses the tumor suppressor P53 in human cancer cells. Immunoprecipitation analysis shows that Piwil2 can directly associate with Signal Transducer and Activator of Transcription 3 (STAT3) protein via its PAZ domain and form a Piwil2/STAT3/c-Src triple protein-protein complex. Furthermore, STAT3 is phosphorylated by c-Src and translocated to nucleus, then binds to P53 promoter and represses its transcription. The present study demonstrated that Piwil2 plays a role in anti-apoptosis in tumor cells possessing P53 as a positive regulator of STAT3 signaling pathway, providing novel sights into roles of Piwil2 in tumorigenesis.

## Introduction

The Argonaute gene family which encode basic proteins that contain both PAZ and Piwi conserved domains, have been reported to induce histone and DNA methylation, mRNA breakdown and inhibition of translation [1], [2]. As a predominantly germline specific clade of Argonaute gene family, Piwi subfamily are found in all animals tested so far and play essential roles in stem-cell self-renewal, gametogenesis and RNA silencing in diverse organisms [3]–[8]. Mutations of three Piwi homologs in mice (miwi, mili and miwi2) respectively cause arrestment of spermatogenesis and male sterility [9]–[11].

The piwil2 gene, alias mili in mouse or Hili in human, is mainly expressed in testis or embryonic cells among normal tissues but widely expressed in tumors, suggesting that Piwil2 may disturb cell division, inhibit apoptosis and play a role as dose-dependent oncogenic fate determinants. However, the underlying mechanism through which Piwil2 involved in tumorigenesis remains largely unknown yet [12], [13].

The tumor suppressor p53 which involved in a number of cellular signaling pathways is known to play an essential role in regulating apoptosis. Loss of P53 function is a common feature of many human cancers [14]–[16]. Though deletions or mutations of P53 have been observed in a great number of tumors, the HeLa cell line possesses wild-type P53 alleles detectable in both mRNA and protein level [17]. Interestingly, Lin et al. reported that in prostate cancer cell lines wild-type but not mutant P53 can significantly inhibit STAT3 activity, whereas it has also been reported that STAT3 can bind to P53 promoter and inhibit the P53 gene transcription rate [18], [19].

STAT signaling pathways activated in response to cytokines and growth factors have been reported to constitutively express in varied tumor-derived cell lines and tumor tissues [20]. STAT3 activation can resist apoptotic machinery relied anti-tumor therapies, and also enhance the growth of tumor cell [18]. Because its activation can mediate oncogenic transformation in cultured cells and tumor formation in nude mice, Stat3 has been classified as an oncogene [21]. Here we present that human piwil2 gene suppresses apoptosis by phosphorylating STAT3 along with c-Src and initiating transcriptional silencing of P53.

## Results

### Piwil2 inhibits P53 involved apoptosis in HeLa Cells

**Figure 1:**
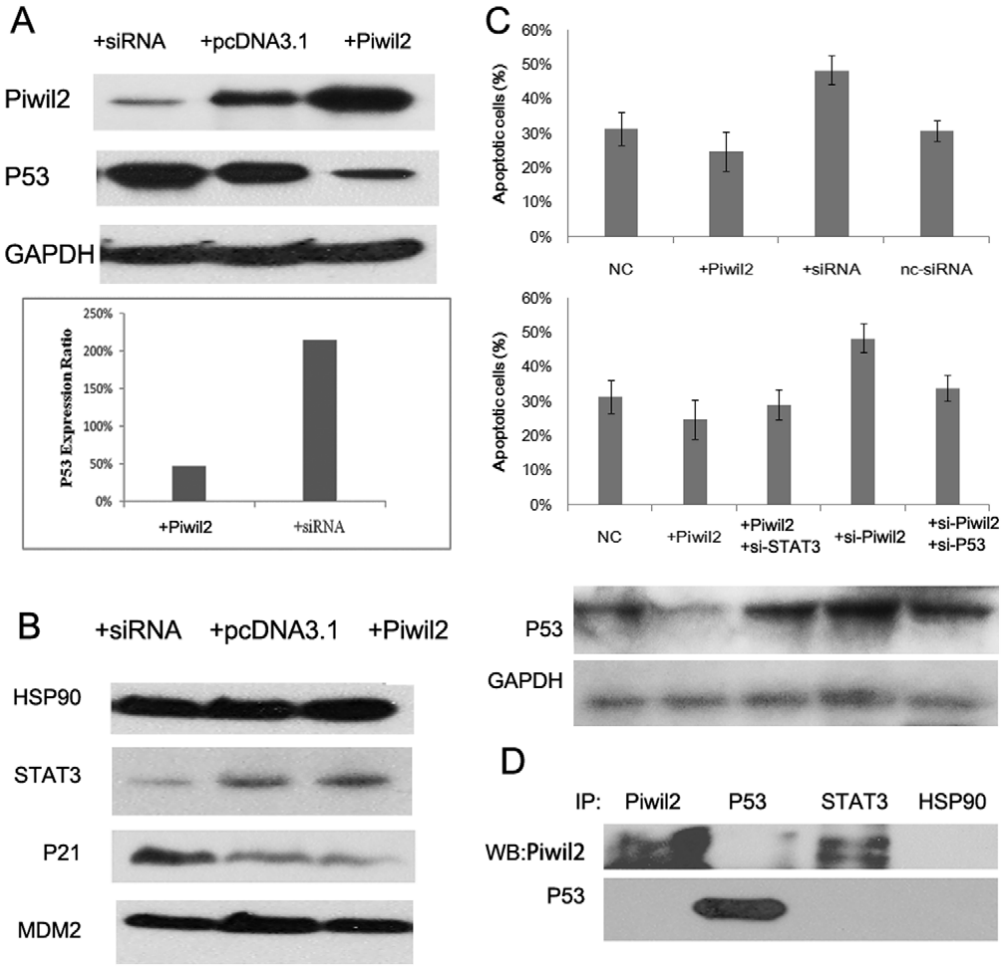
Piwil2 represses P53 and inhibits apoptosis. **A**, P53 expression was significantly repressed by Piwil2. HeLa cells were transfected with same amount of empty plasmid (+pcDNA3.1), pcDNA3.1-Piwil2 (+Piwil2) or Piwil2-specific siRNA (+siRNA), harvested and examined by western blot analysis (upper) and real-time qPCR (lower). Anti-GAPDH antibody and primers were applied as internal control, respectively. **B**, Western blot analysis of expression of HSP90, STAT3, P21 and MDM2 in HeLa cells, suggesting that P21 was downregulated by Piwil2. **C**, Apoptosis analysis of transfected HeLa cells with Annexin V/PI double staining detected by a flow cytometer. Western blot analysis of p53 expression was also performed, suggesting that Stat3 knockdown can rescue the expression of p53 inhibited by overexpressed Piwil2. **D**, Immunoprecipitation analysis showed that STAT3, rather than P53 or HSP90, can directly associated with Piwil2.

Previous studies have reported that Piwil2 is expressed in various tumors and inhibits apoptosis when transfected into embryo fibroblast cells [13]. To investigate the role which human piwil2 protein, alias HILI plays in tumorigenesis and apoptosis in cancer cells, expression vectors and siRNAs were transfected into HeLa Cells. Real-time qPCR and Western blot analysis revealed that there was a significant decrease in P53 expression following the over-expression of Piwil2 in both mRNA and protein level, while its counterpart, Piwil2-knockdowned HeLa cells express a higher level of P53 (Fig. 1A). We also examined several other proteins that have potential roles in tumorigenesis. The results showed that the level of P21 significantly increased when Piwil2 was knockdowned, while slightly decreased when Piwil2 was overexpressed (Fig. 1B).

Fluorescence activated cell sorter (FACS) analysis showed a significant decrease of apoptosis from 31.4% to 24.8%, while its counterpart, piwil2-knockdowned HeLa cells increased to 48.4%. Notably, when P53 specific siRNA was co-transfected into piwil2-knockdowned HeLa cells, apoptosis ratio significantly decreased to 33.9% (Fig. 1C), suggesting that P53 is essential for apoptosis pathway induced by Piwil2-knockdown.

However, immunoprecipitation assay revealed that Piwil2 can not directly associate with P53 (Fig. 1D), suggesting the existence of a potential pathway through which Piwil2 can regulate P53 and inhibit apoptosis in tumor cells.

### Piwil2 suppresses P53 signaling by phosphorylating STAT3

**Figure 2:**
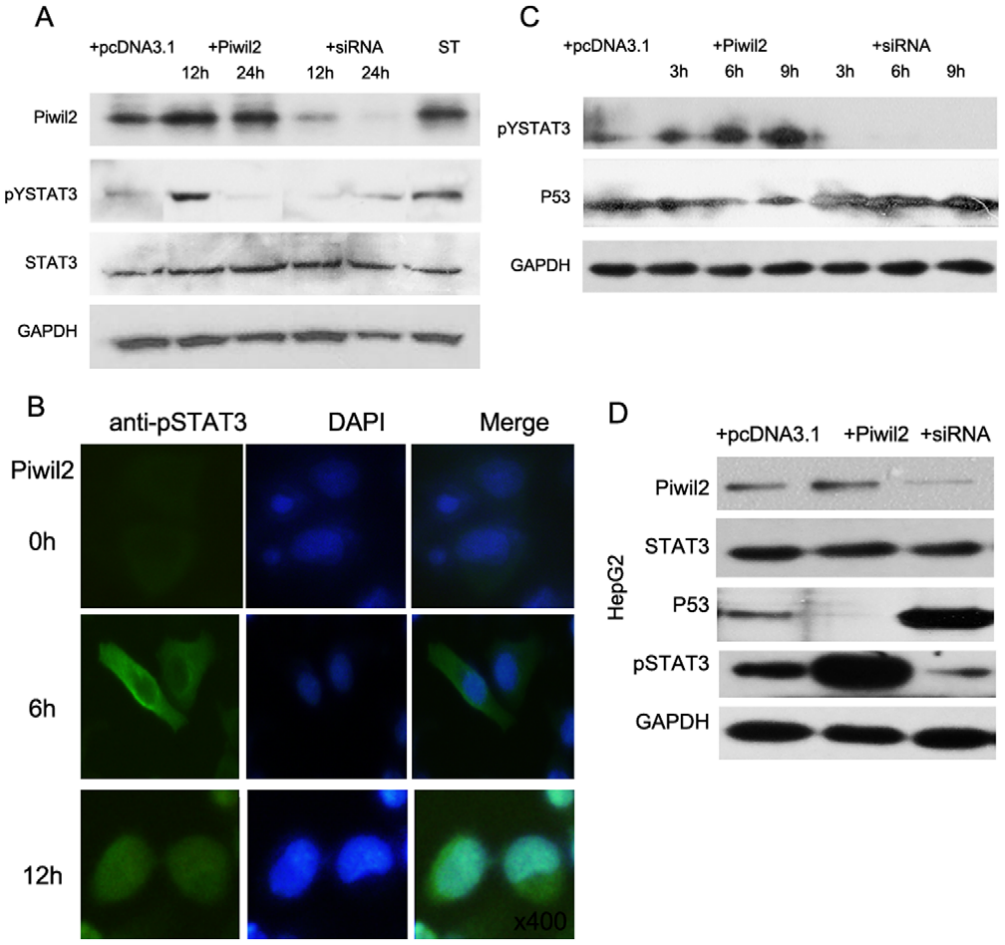
Piwil2 induces phosporylation of STAT3. **A**, Western blot analysis showed that phosphorylation level of STAT3 was significantly increased 12 hours after being transient transfected with Piwil2. However, at 24 hours after transfection, STAT3 phosphorylation level had almost returned to normal. Similar while opposite profile was observed followed transfection of Piwil2-siRNA. Nevertheless, stable transfectants of Piwil2 (ST) showed constantly high STAT3 activity. **B**, pYSTAT3 was subcellular localized in Piwil2 transfected cells by using immunofluorescence. Signal of FITC-labeled secondary antibody was observed significantly increased in cytoplasm 6 hours after transfection, and expanded to nucleus 12 hour after transfection. **C**, time-course analysis showed that STAT3 phosphorylation was notably sensitive to changes in Piwil2 level and responded rapidly, at least 6 hours before P53 regulation. **D**, Western blot analysis revealed that Piwil2 induced STAT3 phosphorylation and repressed P53 in HepG2 cells.

Examination revealed that STAT3 knockdown can block inhibition of P53 expression and apoptosis induced by Piwil2 overexpression (Fig. 1C), indicating that STAT3 protein may play a role between piwil2 and P53 suppression. To determine the underlying mechanism, a western blot analysis was performed to detect the phosphorylation of STAT3, which represents the activation of this transcription factor [22]. A significant increase was observed in Piwil2-overexpressed HeLa Cells 12 hours after transfection. However, after 24 hours, the level of pYSTAT3 resumed its normal level approximately. In contrast, pYSTAT3 level decreased and then recovered after 24 hours in Piwil2-knockdown HeLa cells (Fig. 2A). Immunofluorescence assay also revealed that pYSTAT3 signal increased in cytoplasm 6 hours after pcDNA3.1-Piwil2 transfection (Fig. 2B).

To evaluate the time-course of STAT3 activity and P53 expression, HeLa cells harvested from various time points after transfection was analyzed by western-blotting. The pYSTAT3 level had been boosted just 3 hours after transfection of pcDNA3.1-Piwil2 and continuously increased until 9 hours, whereas the level of P53 showed no more than a slight change until 9 hours. And the transfection of Piwil2-siRNA led to opposite effects in a parallel time-course profile. (Fig. 2C) These results demonstrated that phosphorylation of STAT3 protein occurred earlier than P53 regulation and suggested STAT3 activity may play as an upstream factor of P53 in Piwil2-induced anti-apoptosis.

Furthermore, we examined the level of P53 and pYSTAT3 in another tumor cell line HepG2, revealing that Piwil2 functioned similarly in HepG2 in contrast to HeLa cells (Fig. 2D).

**Figure 3:**
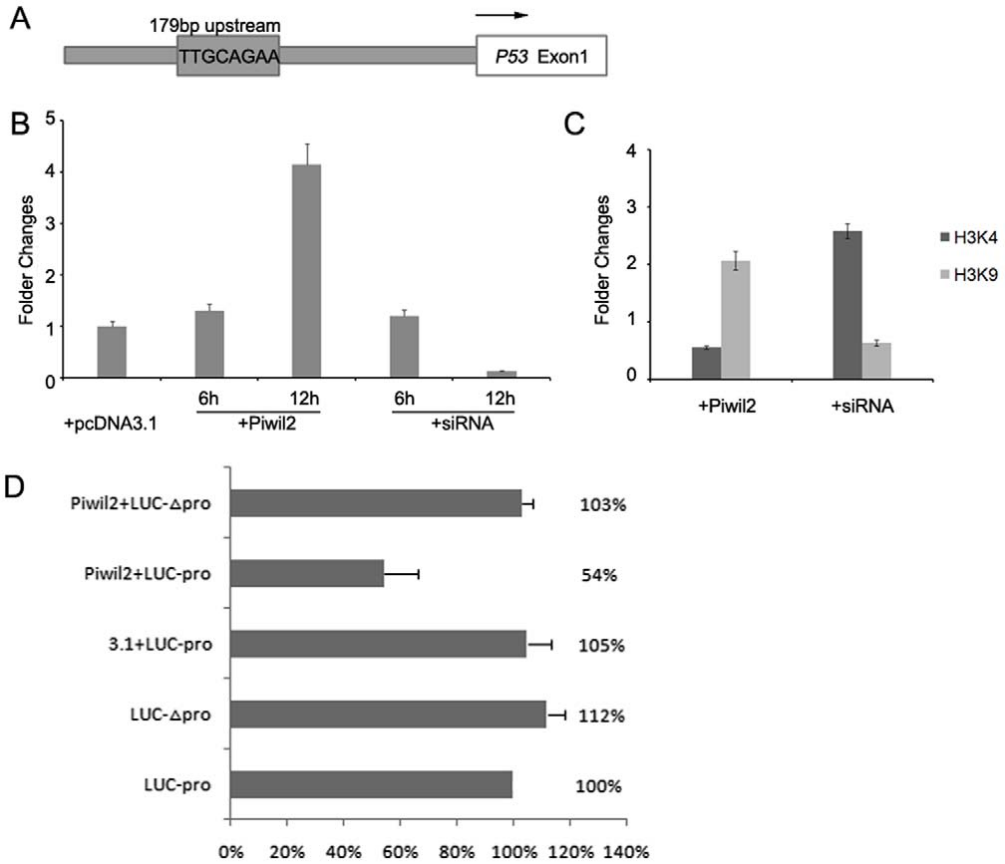
Piwil2 phosphorylated STAT3 binds to P53 promoter. **A**, a potential STAT3 binding site in P53 promoter, 179 bp upstream of the first exon of P53. **B&C**, ChIP assays of chromatin prepared from transfected HeLa cells using the indicated antibodies followed by real-time PCR, which amplified a 127-bp region spanning the Stat3-binding site described above. **B**, STAT3 binding activity to P53 promoter showed significant changes 12 hours after transfection of pcDNA3.1-Piwil2 or siRNA. **C**, histone methylation level of P53 promoter was examined with anti-di-methylation H3K4 (darker) and anti-di-methylation H3K9 (lighter) antibody, respectively. **D**, Piwil2 suppressed the translation of P53 by regulating its promoter. HeLa cells were transfected with the indicated constructs. Relative luciferase activity is the ratio between firefly luciferase and renilla luciferase, adjusted to 100%.

To verify this hypothesis, we determined the interaction between STAT3 protein and P53 promoter using chromatin immunoprecipitation (ChIP) assay followed by Real-time quantitative PCR. Bioinformatic examination revealed that a potential STAT binding site which contains a consensus sequence TT(N_4_)AA or TT(N_5_)AA [19], [23] can be located 179 bp upstream of the noncoding first exon of human P53 (NM_000546.4) (Fig. 3A).

In additional, overexpression of Piwil2 led to an increased level of interaction between STAT3 and P53 promoter 12 hours after transfection. In contrast, knockdown of Piwil2 suppressed such interaction (Fig. 3B). To investgate whether P53 was silenced at transcription level, methylation state of H3K4 and H3K9 in P53 promoter region were analyzed by ChIP method. The results showed that transient transfection of Piwil2 resulted in approximately a 2-folder increase in the methylation state of H3K9 and significant decrease in the methylation state of H3K4, both of which represented the transcriptional silencing of P53. In contrast, knockdown of Piwil2 resulted in significant decrease in the methylation state of H3K9 and increase in the methylation of H3K4 (Fig. 3C).

In order to identify the specific sequence bound by STAT3, the indicated region of p53 gene promoter was mutated and constructed into a pVAX-Luciferase plasmid. Dual-luciferase analysis showed that the translation of mRNAs containing the wildtype but not mutated STAT3 binding site was downregulated by Piwil2 (Fig. 3D).

### Src Kinase is required for STAT3 phosphorylation by Piwil2

**Figure 4:**
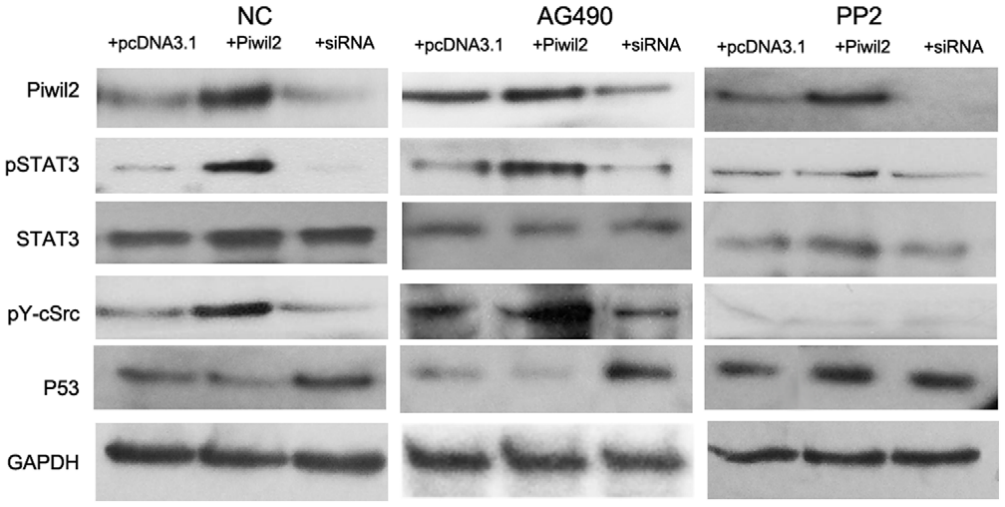
c-Src involved in Piwil2-induced STAT3 phosphorylation. HeLa cells pretreated with JAK-inhibitor AG490 or Src-inhibitor PP2 were transfected with same amount of empty plasmid (+pcDNA3.1), pcDNA3.1-Piwil2 (+Piwil2) or Piwil2-specific siRNA (+siRNA), harvested and examined by western blot analysis. Non-pretreated cells were employed as negative control (NC). The results showed that PP2 rather than AG490 can deplete the activity of c-Src and block the regulation to STAT3 phosphorylation and P53 expression by Piwil2.

To search for potential upstream factors of STAT3, HeLa cells were treated with tyrosine kinase inhibitors before transfection. Western analysis showed that overexpression of Piwil2 still enhanced STAT3 phosphorylation in HeLa cells pretreated with JAK inhibitor AG490. Thus we can rule out JAK family kinases as a phosphate giver. However, in HeLa cells pretreated with Src inhibitor PP2, STAT3 phosphorylation was blocked after overexpression of Piwil2 (Fig. 4A). This result indicated that Src, rather than JAK, played a role in Piwil2-induced STAT3 phosphorylation, which was further confirmed by western blotting using phosphorylated c-Src antibody. Analysis revealed that Y416-phosphorylated c-Src was enhanced 6 hour after pcDNA3.1-Piwil2 transfection, while the level of pY416 c-Src decreased after Piwil2-siRNA transfection (Fig. 4B).

### A triple protein-protein complex is formed when Piwil2 recruits STAT3 to c-Src

**Figure 5:**
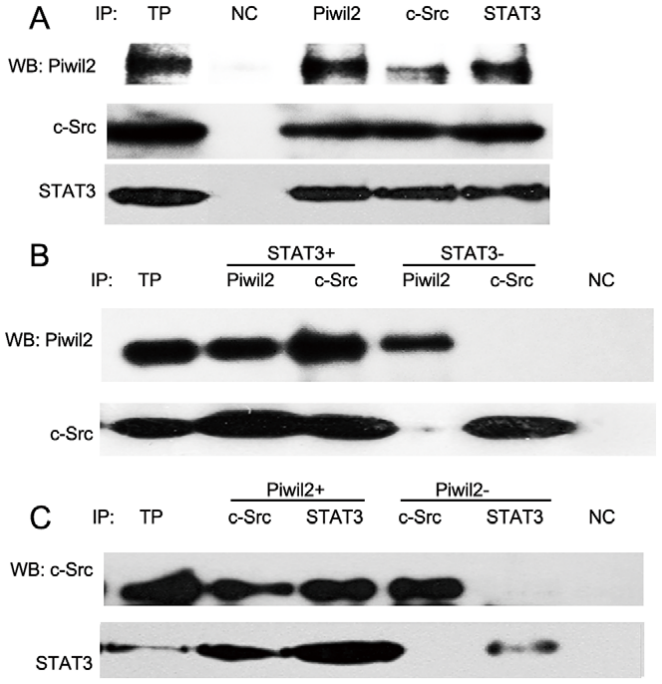
Interaction between Piwil2, c-Src and STAT3. **A**, cell lysates were prepared and subjected to coIP assays. TP, total protein lysates as positive control; NC, protein A+G agarose incubated with total protein lysate to detect nonspecific interaction (negative control, detected with corresponding antibodies). **B**, In STAT3-silenced cells (STAT3−, transfected with STAT3-specific siRNA), the binding between Piwil2 and c-Src was repressed in contrast to lysates of control cells (STAT3+, transfected with nonspecific siRNA). C, the binding between c-Src and STAT3 was repressed in Piwil2-silenced cells (Piwil2-, transfected with Piwil2-specific siRNA).

The effect of Piwil2 on STAT3 phosphorylation was investigated by co-immunoprecipitation (coIP) assay, revealing that the ∼110 kD Piwil2 protein was physically associated with STAT3 and c-Src, while STAT3 was also observed associating with c-Src (Fig. 5A). Meanwhile, the absence of STAT3 blocked the binding between Piwil2 and c-Src (Fig. 5B), whereas Piwil2 knockdown led to decreased c-Src signal detected in STAT3 immunoprecipitates (Fig. 5C). The results suggested that Piwil2 played an essential role in the assocaiation between STAT3 and non-receptor tyrosine kinase c-Src. Thus they formed a triple protein-protein complex and STAT3 was phosphorylated and activated.

**Figure 6:**
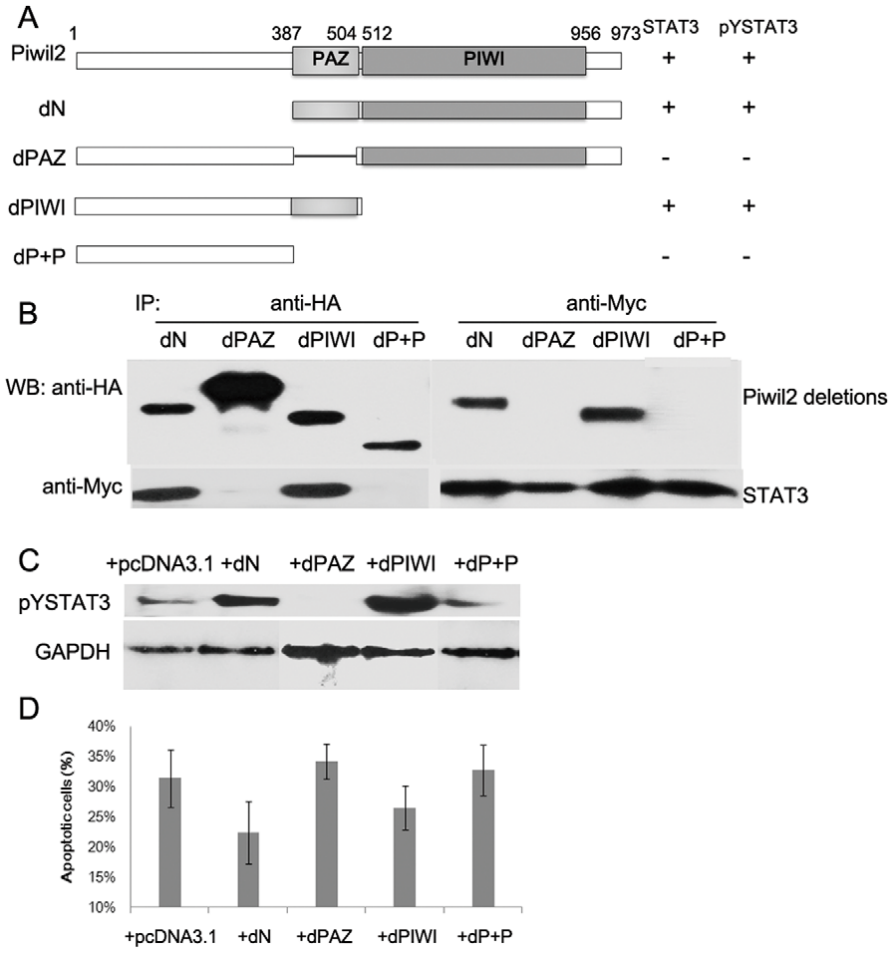
The specificity and domains of Piwil2-Stat3 interaction. **A**, schematic of different Piwil2 deletion constructs. **B**, Piwil2 deletion mutants with HA-tag were respectively co-transfected in HeLa cells with Myc-tagged STAT3, coIP assay revealed that mutants with PAZ domain deletion (pPAZ and pP+P) failed to bind with STAT3. **C**, Western blot analysis showed that PAZ deletion mutants failed to induce phosphorylation of STAT3. **D**, Apoptosis analysis showed that PAZ deletion mutants failed to inhibit apoptosis in transfected HeLa cells.

To further investigate the interaction between Piwil2, STAT3 and c-Src, we constructed a series of Piwil2 deletion mutants to identify the functional domains required for phosphorylating STAT3. Two Piwil2 mutants, one harbored a deletion of PAZ conserved domain while the other lacks both PAZ and PIWI domains, failed to bind with STAT3, while other mutants retained the ability (Fig. 6A and B). Notably, the PAZ-deleted mutants failed to induce STAT3 phosphorylation (Fig. 6C) and inhibit cell apoptosis (Fig. 6D), confirming the potential role of PAZ domain of Piwil2.

## Discussion

It has been reported that Piwil2 transcripts were widely detected in various tumors or cancer cell lines, and played important roles in tumorigenesis and apoptosis inhibition [13], [24]–[26]. However, the mechanism underlying piwil2-mediated tumor development remained unclear yet. To investigate the signaling pathway through which Piwil2 suppresses the apoptosis of cancer cells, we studied the relationship between Piwil2 and its potential target genes. Our research demonstrated that Piwil2 can efficiently activate STAT3 by phosphorylating STAT3 at residue Tyr705 (Fig. 2), which leads to dimerization of STAT3 and downstream targets control [18], [20]. We next examined whether Piwil2 also enhanced STAT3 DNA binding activity. Our results indicated that induced by Piwil2 overexpression, STAT3 DNA binding activity to P53 promoter was significantly increased, leading to epigenetic modification and silencing of P53 gene (Fig. 3B and C). This founding was supported by previous study on roles of STAT3 in regulating P53 by Niu et al [19].

Activation of STAT3 required tyrosine kinases activity, which Piwi family proteins have not yet been reported to possess. JAK/STAT3 pathway is well-known to respond extracellular signal and regulate expression of various genes [27]–[29]. However, AG490, a specific JAK/STAT3 pathway inhibitor, showed little effort in blocking Piwil2-induced STAT3 phosphorylation (Fig. 4A), suggesting that JAK family kinases may not be involved in Piwil2-STAT3-P53 pathway. So we next analyzed another candidate Src, a family of non-receptor tyrosine kinases that has been reported to constitutively activate STAT3 during oncogenic transformation [30]–[33]. Western blot experiments revealed that the overexpression of Piwil2 in PP2-treated HeLa cells failed to increase pYSTAT3 level, while the knockdown of Piwil2 made no significant decrease of pYSTAT3 compared to the control. Further experiments revealed that overexpressed Piwil2 can increase the phosphorylation level on Tyr416 of c-Src, which indicates tyrosine kinase activity of the protein (Fig. 4B).

Immunoprecipitation assay demonstrated that Piwil2, STAT3 and c-Src can physically associated with each other. However, silencing of Piwil2 or STAT3 may lead to significantly repressed binding level between c-Src and the other protein(Fig. 5), suggesting that Piwil2 played an essential role in the association between STAT3 and non-receptor tyrosine kinase c-Src. Thus they formed a triple protein-protein complex and STAT3 was phosphorylated and activated.

**Figure 7:**
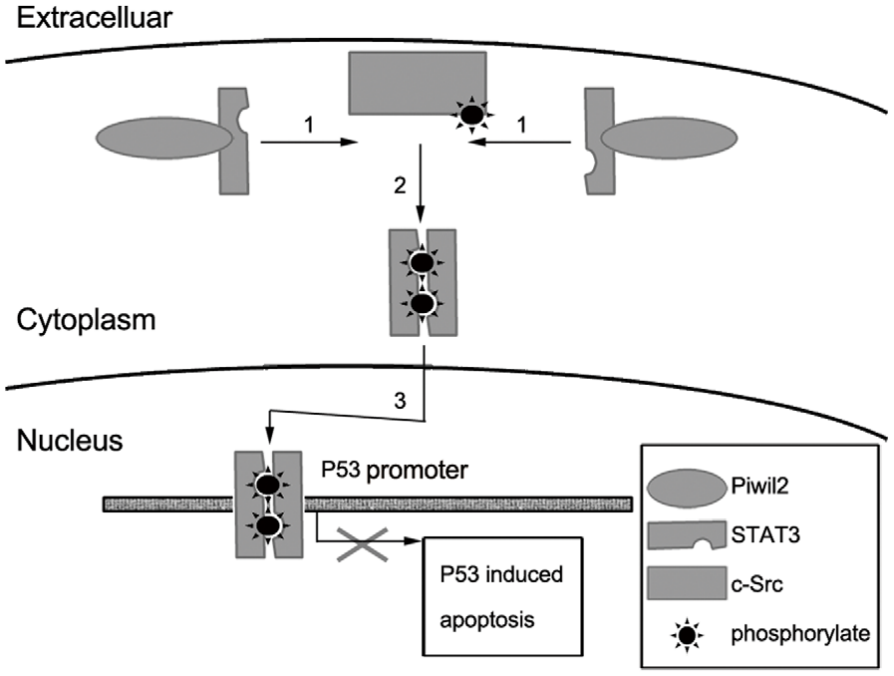
A schematic model for the involvement of Piwil2 in anti-apoptosis in HeLa cells. **1**, Piwil2 binds with monomeric STAT3 and recruited it to c-Src. **2**, STAT3 is phosphorylated by c-Src and then dimerized. **3**, dimeric pYSTAT3 translocates into the nucleus and bind to P53 promoter, repress P53 transcription and thus inhibit apoptosis.

Based on our results and previous finding about STAT and P53, we demonstrated that Piwil2 inhibits P53 involved apoptosis through Src-STAT3 pathway in HeLa cells (Fig. 7). Our immunoprecipitation analysis shows that Piwil2 can directly associate with STAT3 via its PAZ domain and form a Piwil2/STAT3/c-Src triple protein-protein complex. As shown in Figure 1 and Figure 2, our present study shows that the activation of STAT3 phosphorylated by c-Src at residue Tyr705 is significantly increased in Piwil2 overexpressed HeLa cells. The phosphorylated STAT3 proteins dimerize and translocate to nucleus as reviewed by Burdon et al [22]. Furthermore, dimerized STAT3 proteins bind to the promoter region of P53 gene and repress P53 expression.

To study the function of human Piwil2 gene, alias HILI in Hela cells, we analyzed its relationship with P53 and apoptosis. As variant deletions or mutations of P53 have been observed in a great number of tumors, the underlying mechanism of anti-apoptosis may differ among variant cancer cells. However, our results demonstrated that Piwil2 functioned to induce STAT3 phosphorylation and prevent expression of P53 at least in two cell lines (HeLa and HepG2). These investigations will provide a novel insight into roles of Piwil2 in tumor cells.

## Materials and Methods

### Plasmids and siRNAs

The coding sequences of Piwil2, STAT3 and c-Src were amplified by RT-PCR and cloned into the vector pcDNA3.1 (Invitrogen, USA) with an upstreaming Myc- or HA-tag respectively. TRIpure reagent, a gel extraction kit, and a high purity plasmid preparation kit were purchased from Bioteke Corporation (Beijing, China) for total RNA extraction, PCR product purification, and plasmid preparation. The specific siRNAs (described in Table 1) and universal negative control dicer substrate duplex were synthesized and purchased from RiboBio Co. Ltd (Guangzhou, China).

**Table 1:** Sequences of deployed siRNAs.

siRNAs	Sequences
Piwil2 sense	CUA UGA GAU UCC UCA ACU ACA GAA G [24]
Piwil2 antisense	CUU CUG UAG UUG AGG AAU CUC AUA GUU [24]
c-Src sense	AAC AAG AGC AAG CCC AAG GAU dTdT
c-Src antisense	AUC CUU GGG CUU GCU CUU GUU dTdT
STAT3 sense	CAU CUG CCU AGA UCG GCU AdTdT
STAT3 antisense	UAG CCG AUC UAG GCA GAU GdTdT
P53 sense	GAC UCC AGU GGU AAU CUA CdTdT
P53 antisense	GUA GAU UAC CAC UGG AGU CdTdT
Nonspecific duplex	*siR-Ribo™ Negative Control (Ribobio, China)*

The putative STAT3 binding site in p53 gene promoter was obtained and mutated through a PCR method, then cloned into a pVAX-Luciferase plasmid (maintained in our laboratory). The sequences of the PCR primers used are as follows: wildtype forward: 5′- CGC GGA TCC AGC TCT GGC **TT**G CAG AAT TTT CCA C -3′; mutated forward: 5′- CGC GGA TCC AGC TCT GGC **GG**G CAG AAT TTT CCA C -3′; universal reverse: 5′- CCG GAA TTC CCG GAG GAA GCA AAG GAA ATG G -3′.

### Cell culture

Cervical cancer cell line HeLa was maintained in our laboratory [34]; hepatocellular carcinoma cell line HepG2 was obtained from State Key Laboratory of Biotherapy and Cancer Centre of West China Hospital [35]. Both cell lines were maintained in RPMI-1640 medium (Gibco, USA) containing 10% heat-inactivated FBS, 100 U/ml penicillin and 100 µg/ml streptomycin, on 25 cm^2^ culture dishes in a humidified atmosphere containing 5% CO_2_ incubator at 37°C. The cells were passaged by trypsinization every 2–3 days. The transfection was performed with lipofectamine 2000 solution (Invitrogen, USA) according to the manufacturer's protocol and transfected cells were harvested and analyzed 24 hours after transfection, unless stated otherwise. Empty plasmid DNA was applied to transfection as negative control. And in some experiments, stable overexpression transfectants selected by being cultured in medium containing 1 mg/ml of G418 (Solarbio, China) for one month were also examined. To inhibit tyrosine kinase activity, HeLa cells were pretreated with 25 µM AG490 (Sigma-Aldrich, USA) for 24 hours or 10 µM PP2 (Sigma-Aldrich, USA) for 30 minutes. All following experiments were repeated at least three times unless stated otherwise.

### Apoptosis assay

Apoptotic rates were analyzed by a COULTER EPICS XL flow cytometer (Beckman, USA) using an Annexin V-EGFP Apoptosis Detection Kit (Bestbio, Shanghai). Annexin V/PI staining and fluorescence intensity measurements were performed according to the manufacturer's instruction.

### Western Blot Analysis

Harvested cells were lysed in ice-cold universal protein extraction buffer (Bioteke, Beijing) supplemented with protease inhibitor cocktail (Roche, USA) for 30 min. Cell lysates were separated on 4–12% SDS-page gel and transferred to a nitrocellulose membrane. Membranes were blocked in TBS-T Buffer (50 mM Tris-HCl, 150 mM NaCl, 0.1% Tween, PH 7.6) supplemented with 5% nonfat dry milk. The membranes were incubated overnight at 4°C with the indicated primary antibody: rabbit polyclonal anti-Piwil2 (Santa Cruz, USA), rabbit polyclonal anti-STAT3 (Abcam, UK), rabbit polyclone anti-pYSTAT3 (CST, USA), rabbit polyclonal anti-c-Src (Abcam, UK), rabbit polyclonal anti-pY416-c-Src (CST, USA), mouse monoclonal anti-GAPDH (Boster, China), mouse monoclonal anti-HA-tag (CST, USA), and rabbit polyclonal anti-Myc-tag (Santa Cruz, USA), followed by 5 minutes washes in TBS-T for three times and incubation with HRP-labeled secondary antibody (Zhongshan Goldenbridge, China) in TBS-T for 1 hour at RT. The membranes were detected with chemiluminescent HRP substrate (Millipore, USA).

### Immunofluorescence

Transfected cells were fixed with 4% formaldehyde in PBS for 15 min, permeabilized with 0.5% Triton X-100 for 10 min, blocked with 1% BSA for 30 min, incubated overnight at 4°C with primary antibody and finally incubated with FITC-labeled secondary antibody (Zhongshan Goldenbridge, China) for 1 hour at RT. Each step was followed with 5 minutes washes in PBS twice. The prepared specimens were counterstained with 5 µg/mlDAPI for 2 min and observed with a fluoresence microscope (Olympus, Japan).

### Immunoprecipitation

Prepared cell lysates were incubated with 0.8 µg antibody against HA-tag or Myc-tag for 2 hours at 4°C with gentle inverting, then incubated with 20 µl of protein G&A agarose (Beyotime, China) overnight, and precipitated by centrifugation at 12,000 g for 1 min. Complexes were washed four times in ice-cold PBS Buffer (pH 7.4), and electrophoresed on 4–12% SDS-page gel. Western blot detection was carried out as described earlier.

### Chromatin immunoprecipitation (ChIP) assays and real-time PCR

Preparation of chromatin-DNA and ChIP assays were performed as described by the manufacturer's protocol of EZ-Zyme Chromatin Prep Kit (Millipore, USA) and Chromatin Immunoprecipitation Kit (Millipore, USA). Antibody against di-methylated H3K4 and di-methylated H3K9 were purchase from Abcam (UK). Purified DNA was subjected to PCR using primers specific for a 127-bp region spanning the STAT3-binding site in the P53 promoter. The sequences of the PCR primers used are as follows: P53 forward: 5′-ATT CTG CCC TCA CAG CTC TGG CT-3′; P53 reverse: 5′-CCG GAG GAA GCA AAG GAA ATG G-3′; P53 TaqMan: 5′-FAM-CCG CAG TTT CTT CCC ATG CAC CTG-TAMRA-3′. Quantitative PCRs were performed in an iCycler IQ real-time PCR Detection System (BioRad, USA), with a first denaturation step at 94°C for 10 min, followed by 45 cycles comprising denaturation at 94°C for 20 s, annealing at 58°C for 30 s and extension at 72°C for 40 s. Inputs removed before applying antibody were deployed to normalize for differences in the amount of DNA in each PCR.

### Dual-Luciferase Assay

For luciferase analysis, 100 ng plasmid DNA and 100 ng renilla control plasmid were transfected into HeLa cells. Dual luciferase-activity assays were performed 48 hours after transfection according to the manufacturer's directions (Promega). Three independent experiments were set up in every term.
